# The application of in vivo laser confocal microscopy to the diagnosis and evaluation of meibomian gland dysfunction

**Published:** 2008-07-09

**Authors:** Yukihiro Matsumoto, Enrique Adan Sato, Osama M.A. Ibrahim, Murat Dogru, Kazuo Tsubota

**Affiliations:** 1 Johnson & Johnson Department of Ocular Surface and Visual Optics, Keio University School of Medicine, Tokyo, Japan; 2 Department of Ophthalmology, Keio University School of Medicine, Tokyo, Japan

## Abstract

**Purpose:**

To evaluate the morphological changes of the meibomian glands (MG) in patients with meibomian gland dysfunction (MGD) compared to normal subjects by in vivo confocal microscopy and to investigate the relation of these changes to the clinical ocular surface findings and tear functions.

**Methods:**

Twenty MGD patients and 15 normal subjects were recruited into this prospective study. Patients and controls underwent slit lamp examinations, tear film break-up time (BUT) measurements, fluorescein and Rose-Bengal stainings, Schirmer test I without anesthesia, tear evaporation rate assessment (TEROS), tear film lipid layer interferometry (DR-1), transillumination of the lids (meibography), MG expressibility test, and in vivo laser confocal microscopy of the lids (HRTII-RCM).

**Results:**

The BUT, DR-1 tear film lipid layer interferometry grades, fluorescein and Rose-Bengal staining scores, MG drop out grade in meibography, and MG expressibility grades were significantly worse in MGD patients compared to normal controls (p<0.05). The severity of both MG dropout and MG expressibility related significantly with the BUT, DR-1 grades, and TEROS (p<0.05). The mean density of acinar units of MGs as measured by HRTII-RCM was significantly lower in MGD patients (47.6±26.6/mm^2^) than in control subjects (101.3±33.8/mm^2^; p<0.05). The mean acinar unit diameter as determined by HRTII-RCM was significantly larger in MGD patients (98.2±53.3 μm) than in controls (41.6±11.9 μm; p<0.05). Both the density and diameter of MG acinar units related significantly with the severity of MG dropout and MG expression grades (p<0.05).

**Conclusions:**

In vivo confocal microscopy can effectively demonstrate the morphological changes of the MG in patients with MGD. Glandular acinar density and acinar unit diameter seemed to be promising new parameters of in vivo confocal microscopy, which is significantly related to the clinical ocular surface and tear function findings of MGD.

## Introduction

Meibomian glands (MG) are holocrine lipid excreting glands embedded in the tarsal plate of the upper and lower lids. Each MG comprises multiple acini connected by a long common central duct running throughout the entire length of the gland. Epithelial cells comprising the acini synthesize and release lipids into the central ducts, which are then excreted onto the ocular surface. Lipids excreted by MGs form the superficial layer of the tear film and are thought to retard tear evaporation and function as lubricants for the eyelids during blinking [1].

Meibomian gland dysfunction (MGD) is a term that is mainly used to describe obstructive MG disease. MGD is a major cause of dry eyes and has been reported to result in the alteration and/or reduction of lipid secretions, leading to increased tear evaporation, decreased tear stability, loss of lubrication, and damage to the ocular surface epithelium [1].

MGD is one of the most common disorders encountered in ophthalmic practices [2]. Hom et al. [3] reported the prevalence of MGD to be 38.9%. Molinari and Stanek [4] found the prevalence to be 33% in patients younger than 30 years and 71.7% in individuals 60 years or older.

It is important to be able to classify and quantify MGD for diagnostic reasons and to observe effects of treatment. Several methods are available to assess MG status. Simple clinical tests include grading the clinical features of MGD and grading the expressibility and quality of the meibomian oil. Expressibility of meibomian secretion can be performed with digital eyelid pressure ab externo by slit lamp with assessment placed on the viscosity or the clarity of the lipids expressed [5-7].

One of the specialized methods is the measurement of oil levels by meibometry, which involves blotting of lipids from the lower central lid margin onto a plastic tape [8-11]. Recently, such biomicroscopic techniques have evolved allowing for the in vivo imaging of the structure of MGs. Meibography is one that involves transillumination of the MG through the lids using an illuminated probe placed on the skin along the length of the lid. The lid is everted, and the glandular morphology can be photographed with or without the aid of near infrared light [6,12,13]. The absence or dropout of glands over the entire lid length is used as a quantitative measure of gland loss.

Confocal microscopy is a new emerging non-invasive technology, which is useful as a supplementary diagnostic tool for in vivo assessment of the histopathology of many ocular surface diseases and anterior-segment disorders including the in vivo examination of the bulbar and palpebral conjunctiva and the MG [14-20]. In this study, we used confocal microscopy, based on new diagnostic parameters, to evaluate the morphological changes of the MG in patients with MGD and compared the results with those of healthy control subjects. We also investigated the relation of these morphological changes and parameters to the clinical ocular surface findings and tear functions.

## Methods

### Subjects and examinations

In this single center, prospective clinical study, 40 eyes from 20 patients with MGD (MGD group: 14 women and 6 men; Mean age: 65.9±15.5 years; range: 30–99 years) and 30 eyes of 15 healthy control subjects (Control group: 8 women and 7 men; Mean age: 56.8±13.6 years; range: 38–77 years) were recruited. None of the MGD patients and the controls had a history of Sjögren syndrome, Stevens-Johnson syndrome, chemical, thermal, and radiation injury, or had undergone any ocular surgery or procedure that would create an ocular surface problem. Subjects in this study also did not have a history of systemic drug or contact lens use. At ocular examination, particular attention was paid to lids margins, tarsal and bulbar conjunctiva, and corneas. MGD was assessed by careful slit lamp examination of the glandular orifices, mucocutaneous junction changes, and digital expression of the meibomian lipids. All examinations were performed by a single investigator (Y.M.). The same physician pressed gently on the upper and lower eyelids to express the meibomian lipids. Meibum viscosity was graded as described by Shimazaki et al. [6]. Only patients with simple MGD who met all of the following criteria were included in this study. The criteria for the diagnosis of simple MGD were described as follows: 1) occluded MG orifices; 2) cloudy or inspissated glandular secretion with lack of clear meibum secretion following the application of moderate digital pressure on the tarsus of the upper and lower eye lid; 3) presence of keratinization or displacement of the mucocutaneous junction; 4) absence of inflammatory lid disease such as blepharitis as well as inflammatory skin disorders such as atopic dermatitis, seborrhea sicca, and acne rosacea; 5) absence of a history of cicatricial eyelid and conjunctival diseases such as trachoma, erythema multiforme, ocular cicatricial pemphigoid, and chemical, thermal, or radiation injury; and 6) absence of excessive meibomian lipid secretion (seborrheic MGD). All subjects in this study gave their informed consent. The examination procedures were reviewed by the ethics board and approved at Keio University School of Medicine.

Both eyes of each patient and control underwent tear film lipid layer interferometry, tear evaporation rate measurements from the ocular surface (TEROS), slit lamp examinations including tear film break-up time (BUT), fluorescein and Rose-Bengal stainings of the ocular surface, Schirmer test І without anesthesia, in vivo laser confocal microscopy, transillumination of the lids with a transilluminator (meibography), and the meibomian gland expressibility test in this respective order.

### Tear evaporation rate

We measured the TEROS with a quartz crystal humidity sensor (Kao Analytical Research Center, Tochigi, Japan) [21,22]. The temperature and humidity of the examination room were maintained at a range from 20 °C to 25 °C and from 30% to 50%, respectively.

### Tear film lipid layer interferometry

Tear film lipid layer interferometry (DR-1; Kowa Co., Tokyo, Japan) was also performed to evaluate the status of the tear film in all subjects. DR-1 interferometry observes the specular reflected light from the tear surface [23,24]. The classification of tear lipid layer patterns and gradings has been described previously [23]. Briefly, the DR-1 images were classified from grade 1 to 5 as follows: Grade 1-somewhat gray color and uniform distribution; Grade 2-somewhat gray color and non-uniform distribution; Grade 3-a few colors and non-uniform distribution; Grade 4-many colors and non-uniform distribution; and grade 5-corneal surface partially exposed [23].

### Tear film stability evaluation

The standard tear film BUT measurement was performed after instillation of 2 µl of 1% fluorescein preservative-free solution in the conjunctival sac with a micropipette. The patients were instructed to blink several times for a few seconds to ensure adequate mixing of the dye. The interval between the last complete blink and appearance of the first corneal black spot in the stained tear film was measured three times, and the mean value of the measurements was calculated. A BUT value of less than 5 s was considered abnormal.

### Tear quantity evaluation

For further evaluation of tears, the standard Schirmer test І without topical anesthesia was performed. The sterilized strips of filter paper (Showa Yakuhin Kako Co. Ltd., Tokyo, Japan) were placed in the lateral canthus away from the cornea and left in place for 5 min. Readings were recorded in millimeters (mm) of wetting for 5 min. A value of less than 5 mm was considered abnormal.

### Ocular surface examinations

Preservative free solution (2 μl) consisting of 1% fluorescein and 1% Rose-Bengal dye were applied to the conjunctival sac with a micropipette as described before [25]. The Rose-Bengal and fluorescein staining scores of the ocular surface ranged between 0 and 9 points. The van Bijsterveld scoring system was used for Rose-Bengal staining. Briefly, the ocular surface was divided into three zones: nasal conjunctival, corneal, and temporal conjunctival areas. A staining score between 0 and 3 points was employed in each zone with the minimum and maximum total staining scores ranging between 0 and 9 points. The presence of scarce punctate staining received 1 point. The presence of denser staining not covering the entire zone received 2 points, and the presence of Rose-Bengal staining over the entire zone received 3 points. In fluorescein staining, the cornea was divided into three equal upper, middle, and lower zones. Each zone had a staining score ranging between 0 and 3 points with the minimum and maximum total staining scores ranging between 0 and 9 points. Likewise, the presence of scarce staining in one zone was scored as 1 point whereas punctate staining covering the entire zone was scored as 3 points.

### Meibography

Transillumination examination (meibography) was performed by using a transilluminator (Medicospot; MS-H, Neitz Instruments Co. Ltd., Tokyo, Japan). Transillumination of the MG through the lids was achieved by using an illuminated probe placed on the skin of the lower eye lid, which was everted, and the glandular morphology could be photographed along the entire length of the lower lid by the slit lamp including nasal, temporal, and central lid areas. This allowed quantification of the meibomian glandular rows and their loss. Loss of visible MG structure (“gland dropout”) revealed by meibography was considered as evidence of obstructive MGD since this finding has been reported to be a good parameter for obstructive MGD-associated ocular surface disease [6,12,13]. The degree of MG dropout was scored as described previously: Grade 0-no gland dropout; Grade 1 dropout-loss of less than half of the glandular structures; and Grade 2 dropout-loss of more than half of the glandular structures [6,13].

### Meibomian gland expressibility

After digital pressure application on the upper lid tarsus [5-7], the viscosity and degree of meibum expression was evaluated semi-quantitatively according to Shimazaki grading as follows: Grade 0-clear meibum expressed easily; Grade 1-cloudy meibum expressed with mild pressure; Grade 2-cloudy meibum expressed with more than moderate pressure; and Grade 3-meibum cannot be expressed even with intense pressure [6].

### In vivo laser confocal microscopy

In vivo laser confocal microscopy was performed on all subjects with a new generation confocal microscope, the Rostock Corneal Software Version 1.2 of the HRTII-RCM (Heidelberg Retina Tomograph II- Rostock Cornea Module, Heidelberg Engineering GmbH, Dossenheim, Germany) [17]. After topical anesthesia with 0.4% oxybuprocaine, the subject’s chin was placed on the chin rest. The objective of the microscope was an immersion lens covered by a polymethylmethacrylate cap (Tomo-Cap; Heidelberg Engineering GmbH, Dossenheim, Germany). Comfort gel (Bausch&Lomb GmbH, Berlin, Germany) was used as a coupling agent between applanating lens cap and the eye lid. After the eyelid was everted, the center of the Tomo-Cap was applanated onto the palpebral conjunctiva by adjusting the controller, and the digital images of the underlying MG could be observed on the computer screen. When the first superficial conjunctival cells were visualized, the digital micrometer gauge was set at zero, and then by pressing on the foot pedal, sequence images were recorded by a charge-coupled device (CCD) color camera (maximum 30 frames/s) while gradually moving the focal plane into the subconjunctival tissue until the glandular structures were visualized. MGs were scanned while moving the applanating lens from the lids margins toward the fornix with minute vertical movements. MGs were also scanned while moving the applanating lens along the entire lid length with minute horizontal movements. The length of a single confocal microscopy examination session was approximately 10 min. None of the subjects complained of discomfort nor any adverse effect was observed after an examination in this series.

The laser source employed in the HRTII-RCM was a diode laser with a wavelength of 670 nm. The two-dimensional image sizes were 384x384 pixels with a 400x400 μm field of view. Optical resolution was 2 μm vertically and 4 μm horizontally, and the digital resolution was 1 μm/pixel vertically and horizontally. The instrument also gave the focal distance of the image obtained, thereby allowing the determination of the depth in microns. We devised two new parameters, acinar unit diameter and density, to evaluate the morphological changes in the MGs in this study. The acinar unit diameter was measured with the internal software in micrometers as the longest axis of the acinar unit according to the protocol of this study. Clearly visible acinar units were all counted in a 400x400 μm frame, and the acinar density was described as the number of units/mm^2^. We calculated the mean diameter and density of glandular acinar units from three randomized non-overlapping high quality digital images of the nasal, middle, and temporal lower eyelid (total of nine images per eyelid).

### Statistical analysis

All data are shown as the mean±standard deviation (SD). The Mann–Whitney test was applied to test the statistical differences between BUT, Schirmer test І, TEROS, the acinar unit diameter, and density of acinar units. The Mann–Whitney U test was used to study the differences between the fluorescein staining score, Rose-Bengal staining score, MG dropout, and meibum expression grades. Sex differences between the groups were evaluated by using the χ^2^ test. Inter-group differences were evaluated by using the one-way ANOVA test. The differences were considered statistically significant if the p values were less than 0.05.

## Results

There were no significant age and gender differences between normal controls and MGD patients (p>0.05).

### Tear functions and ocular surface staining scores

Comparisons of tear functions, ocular surface findings, and age and gender characteristics between normal controls and MGD patients are shown in Table 1. The mean Schirmer test value was not significantly different in normal controls (12.7±9.6 mm) when compared to the mean value in MGD patients (9.3±7.2 mm; p>0.05).

**Table 1:** Comparison of age, gender, tear quantity, tear stability, lipid layer interferometry, tear evaporation rate, vital staining scores and clinical meibomian gland status between normal controls and MGD patients.

	**Controls (n=15)**	**MGDs (n=20)**	**p value**
Age (years)	56.8±13.6	65.9±15.5	p=0.074
Gender (% of female)	53.3	70.0	p=0.51
Schirmer test (mm)	12.7±9.6	9.3±7.2	p=0.11
BUT (s)	8.5±3.7	4.9±2.4	p=0.0001
DR-1 grading	2.3±0.8	3.6±0.8	p=0.0001
TEROS (10^−7^ g/cm^2^/s)	4.30±3.82	6.37±3.72	p=0.029
FS score (points)	0.5±0.9	2.4±2.8	p=0.0001
RB score (points)	0.2±0.6	1.1±1.7	p=0.0067
MG expressibility grading	0.3±0.5	2.2±0.9	p=0.0001
MG dropout grading	0.4±0.6	1.6±0.5	p=0.0001

Note the absence of significant age and sex differences between the controls and MGD patients. The Schirmer test value (tear quantity) was also not significantly different in MGD patients when compared to controls. Note that the mean tear stability (BUT), DR-1 lipid layer interferometry grade, tear evaporation rate, vital staining scores (FS and RB scores), MG expressibility, and drop out grades were significantly worse in patients with MGD when compared with controls. Data are given as mean±SD. p values denote the statistical comparison between controls and MGDs. MG=meibomian gland, MGD=meibomian gland dysfunction, BUT=tear film break-up time, TEROS=tear evaporation rate from ocular surface, DR-1=tear film lipid layer interferometry, FS=fluorescein, RB=Rose Bengal.

The BUT values were significantly lower in MGD patients (4.9±2.4 s) when compared to normal controls (8.5±3.7 s; p<0.05). Both the DR-1 interferometry grades and the mean TEROS were significantly higher in MGD patients when compared to controls (p<0.05). The mean fluorescein and Rose-Bengal staining scores were significantly higher in MGD patients (2.4±2.8, 1.1±1.7, respectively) when compared with the controls (0.5±0.9, 0.2±0.6, respectively; p<0.05).

### MG drop out grades (meibography)

**Figure 1:**
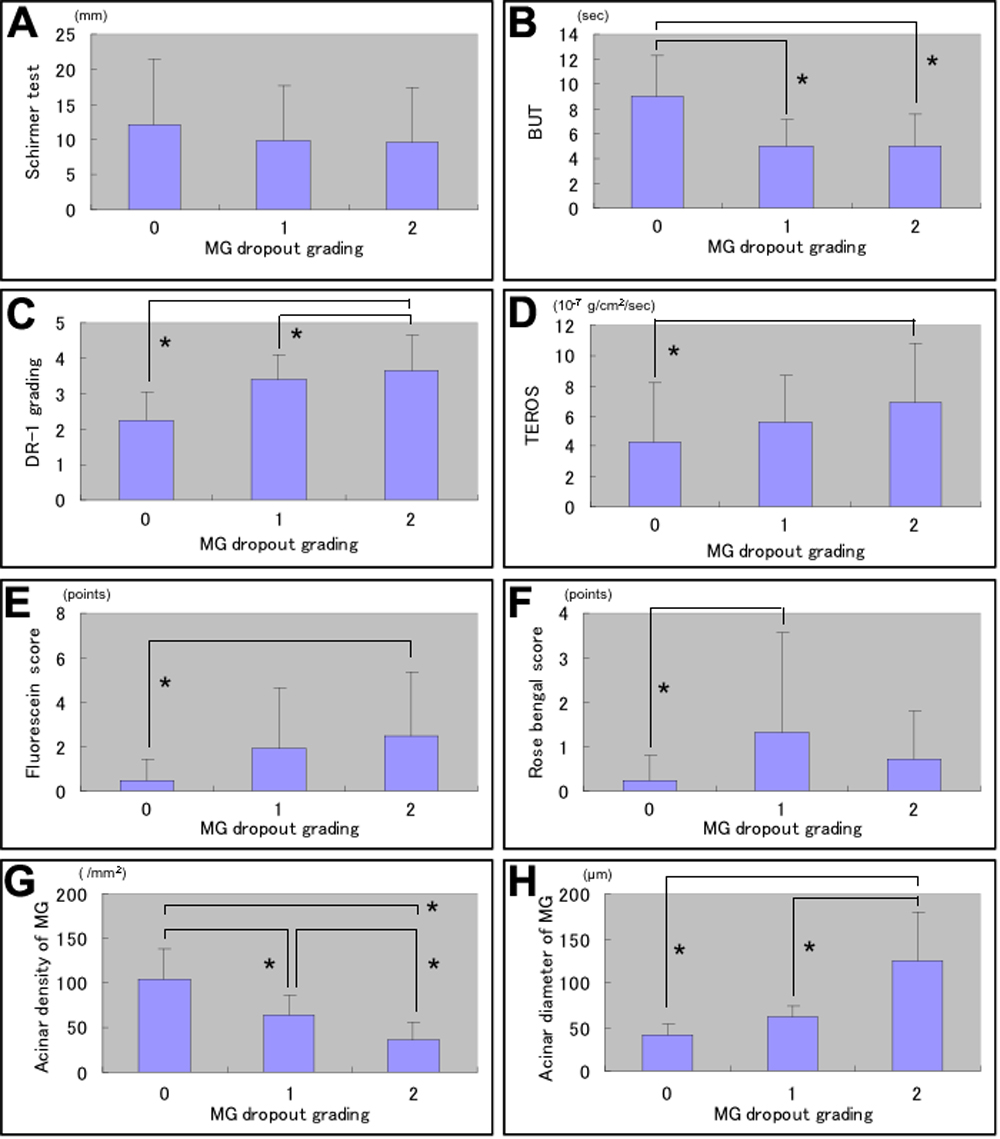
Relation of meibomian gland (MG) dropout grades with tear functions, ocular surface disorder, MG acinar unit density and diameters. **A**-**H**: The MG drop out grade (severity) showed significant relations with tear functions including BUT, DR-1 tear film lipid layer interferometry grades, TEROS, and fluorescein and Rose-Bengal staining scores (p<0.05) except the mean Schirmer test value (p>0.05; **A**-**F**). Both the mean acinar unit density and diameter showed significant relations with MG drop out grades (p<0.05; **G**-**H**).

None of the MGD patients had grade 0 drop out changes in meibography. The mean MG dropout grade as observed with meibography in MGD patients was significantly higher compared to normal controls (p<0.05) as shown in Table 1. The MG dropout grade (severity) showed significant relations with tear functions including BUT, DR-1 tear film lipid layer interferometry grades, TEROS, fluorescein, and Rose-Bengal staining scores (p<0.05) each except the mean Schirmer test value (p>0.05; Figure 1A-F).

**Figure 2:**
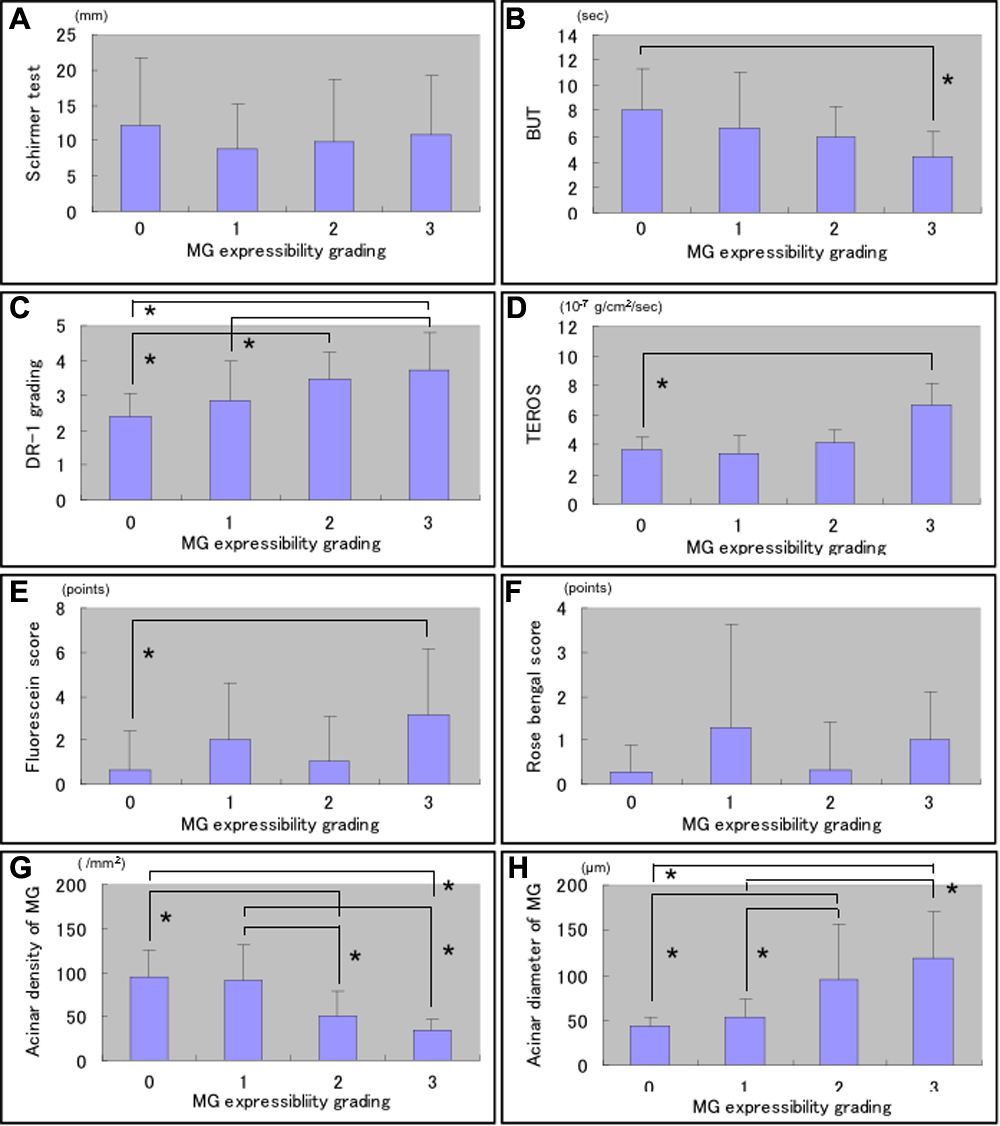
Relation of meibomian gland (MG) expressibility grades with tear functions, ocular surface disorder, and MG acinar unit density and diameters. The MG expressibility grade also showed significant relations with the mean BUT values, DR-1 interferometry grades, TEROS values and fluorescein scores (p<0.05) except the mean Schirmer test and Rose Bengal staining scores (p>0.05; **A**-**F**). Both the density and diameter of acinar units were significantly related with the MG expressibility grades (p<0.05; **G** and **H**).

None of the MGD patients had grade 0 expressibility in this study. The mean MG expression grade was also significantly higher in MGD patients than in controls (p<0.05) as shown in Table 1. The MG expressibility grade also showed significant relations with the mean BUT values, DR-1 interferometry grades, TEROS values, and fluorescein scores except the mean Schirmer test and Rose Bengal staining scores (p>0.05; Figure 2A-F).

### In vivo laser confocal microscopy meibomian gland observations

**Figure 3:**
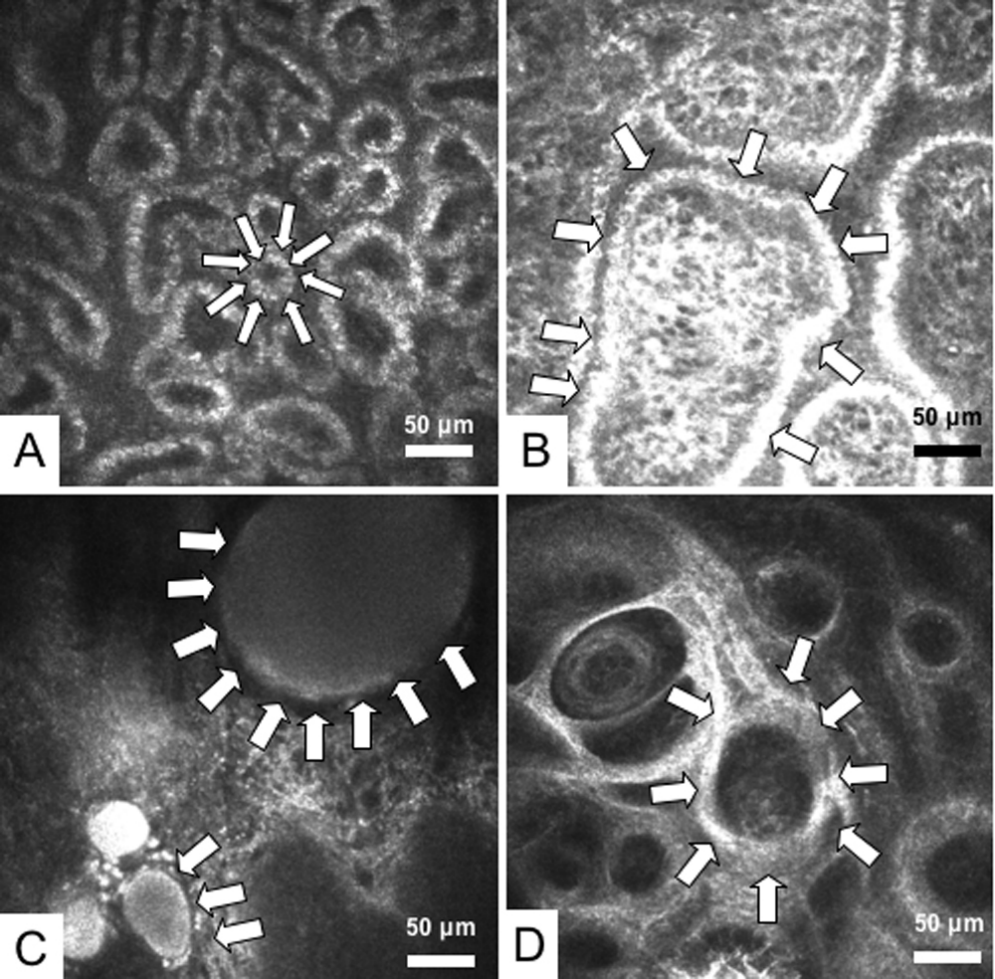
Meibomian gland (MG) images observed by in vivo laser confocal microscopy. **A**: One of the MG images from a representative 45-year-old male normal control subject is shown. White arrows depict a typical acinar unit. Note the presence of numerous and compact acinar units. Mean acinar unit densities and diameters were calculated from a total of nine confocal images obtained from the lower lid of the subject. The mean acinar unit density calculated was 112/mm^2^, and the mean acinar unit diameter was 46 μm. The diameter of the acinar unit depicted by the arrows is 35 μm. **B**: One of the representative MG images from a 66-year-old female patient with MGD is shown. Note the enlargement of acinar unit (outlined by white arrows) due to inspissation of meibum secretion. The MG drop out grade was 1, and expressibility grade was 1. Mean acinar unit densities and diameters were calculated from a total of nine confocal images obtained from the lower lid of the subject. The mean acinar unit density calculated from the overall images was 69/mm^2^, and the acinar unit diameter was 72 μm. Note that acinar units might show considerable enlargement. The diameter of the acinar unit depicted by the arrows is greater than 250 μm. **C**: One of the representative MG images from a 54-year-old female patient with MGD is shown. The patient had advanced disease with grade 2 MGD and the meibum secretion could not be expressed due to orifice obstruction (MG expressibility grade 2). Note the remarkable dilatation of acinar units with inspissation of meibum secretions (white arrows). **D**: Representative MG image from a 78-year-old female patient with advanced MGD is shown. The MG drop out grade was 2, and the MG expressibility grade was 3. Note the atrophy in the glands with extensive periglandular fibrosis (white arrows).

The mean acinar unit density was 47.6±26.6/mm^2^ in MGD patients. This value was significantly lower than the mean value measured in normal controls (101.3±33.8/mm^2^; p<0.05) as shown in Table 2. The mean acinar unit diameter was significantly larger (98.2±53.3 μm) in MGD patients than in controls (41.6±11.9 μm; p<0.05). Both the mean acinar unit density and diameter showed significant relations with MG dropout grades (p<0.05; Figure 1G-H). Likewise, both the mean acinar unit density and diameter also showed significant relations with MG expressibility grades (p<0.05; Figure 2G-H). In vivo laser confocal microscopy could effectively demonstrate the glandular acinar units in healthy control subjects as shown in Figure 3A. In vivo laser confocal microscopy images also disclosed morphological alterations in patients with MGD including the enlargement of glandular acinar units due to inspissation of meibum secretions (Figure 3B,C) and glandular atrophy with periglandular fibrosis (Figure 3D).

**Table 2:** Comparison of acinar unit density and diameter of meibomian glands in normal controls and MGD patients.

	**Controls**	**MGDs**	**p value**
Acinar unit density (/mm^2^)	101.3±33.8	47.6±26.6	p=0.0001
Acinar unit diameter (μm)	41.6±11.9	98.2±53.3	p=0.0001

Note that the mean acinar unit density was significantly lower in patients with MGD when compared with the control subjects. The mean acinar unit diameter was significantly longer in MGD patients than in normal controls. Data are given as mean±SD. p-values denote the statistical comparison between controls and MGDs. MG=meibomian gland, MGD=meibomian gland dysfunction.

## Discussion

MGs secrete lipids into the preocular tear film. These lipids function as a barrier to the inward movement of skin surface lipids, make the eyelid margin hydrophobic, reduce evaporation of tears, and lubricate the ocular surface to provide a clear optical image [13].

MGD is characterized by the inspissation of meibomian lipids, resulting in hyposecretion of lipids into tears. MGD is one of the major causes of ocular discomfort and abnormalities of the ocular surface [1,13]. Reported ocular surface and tear function abnormalities associated with MGD include an increase of TEROS and osmolarity of tears, damage to the ocular surface epithelium as evidenced by increased ocular surface vital staining scores, decrease in conjunctival goblet cell density, increased MG drop out, and decreased meibum expressibility [1,26]. It is possible to evaluate the glandular function and ocular surface status in MG disease by slit lamp observation of the morphological changes in the lid margin including the gland secretion expressibility, BUT measurements, lipid layer interferometry, meibometry and biochemical lipid analyses, ocular surface vital stainings, tear evaporation rate measurements, and meibography [1,11].

In accordance with previously published reports, we confirmed the presence of an ocular surface disease state, which was characterized by shorter BUT, higher lipid layer interferometry grades, TEROS, and higher ocular surface vital staining scores in our MGD patients when compared to healthy control subjects. It was harder to express the meibomian secretions in the patients of the current series as evidenced by the significantly higher expressibility grades. Meibography disclosed a significantly higher level of glandular drop out in our patients compared to controls. Eyes with higher MG drop out grades had significantly worse tear stability, higher tear film lipid layer interferometry grades indicating lipid layer abnormality, higher TEROS and ocular surface vital staining scores. Since glandular drop out may be expected to be associated with lesser meibomian secretions and their expressibility, we also checked the meibum expressibility as described by Shimazaki et al. [6] in a previous report. Similarly, higher grades of expressibility, which indicate comparably more advanced obstruction of the glands, were associated with significantly worse tear stability, higher tear evaporation, and ocular surface epithelial damage. These findings reconfirmed the Shimazaki [6] report that ocular surface and tear function disorders tended to be more severe in eyes of patients with significant gland obstruction and MG drop out.

To further investigate the morphological alterations in the MGs, we employed in vivo laser confocal microscopy and devised two new confocal microscopy based diagnostic parameters, glandular acinar unit density and acinar unit diameter, for the first time in the literature, which we believed might have had a relation with the morphological observations of the MGs such as the MG drop out and MG expressibility grades. To our surprise, eyes with higher MG drop out grades in meibography had significantly lower MG acinar unit densities in confocal microscopy. Likewise, higher drop out grades were associated with significant enlargement of acinar unit diameter, which suggested inspissation of meibomian secretions and inability of the glands to deliver lipids into the tear film. Similarly, patients with higher MG expressibility grades (i.e., patients in whom the MG secretions could not be expressed or could be expressed with difficulty) had significantly lower acinar unit densities and longer acinar unit diameters in confocal microscopy. Confocal microscopy could effectively demonstrate the build up of lipids in the glands in patients with higher gland drop out or expressibility grades as well as the engorgement and dilatation of acinar units. We believe that these observations also explain the previously reported histopathological changes in human MG disease from meibomian glands obtained postmortem [27]. It has been reported in humans and animal models of MGD that hyperkeratinization of ductal epithelium results in the shedding of keratinized material into the duct, which may lead to obstruction of glandular orifices and a build up of glandular secretions with a change in lipid composition [27-30]. The changes in lipid composition have been reported to increase the melting point, leading to an increase in lipid viscosity and further inspissation of the glandular secretions [27]. These events have been suggested to be followed by cystic dilatation of acini and/or ducts [27,28]. The pressure from the accumulating secretions might very well induce acinar atrophy. In this study, confocal microscopy disclosed similar acinar atrophy changes in accordance with previous histopathological reports [27]. The atrophic acinar units appeared as small irregular structures with periglandular fibrosis compared to normal and round acini observed in healthy control subjects.

Acinar unit density and diameter seem to be two promising new confocal microscopy parameters, which we believe will aid in the diagnosis and evaluation of MG disease. Although statistically not significant, it should be noted that the mean age in patients with MGD was higher than the control subjects, which might have very likely affected the results of this study. Recruitment of elderly control subjects with normal gland morphology and functions is a challenging issue due to the age related nature of MGD. Future studies on age matched subjects would certainly eliminate such biases. Future prospective studies on a larger number of patients and healthy controls should also establish the sensitivity, specificity, and cut-off values for these diagnostic parameters, which may also find applications in the assessment of treatment outcomes in patients with MGD. The status of meibomian glands and changes of these parameters in MGD associated with inflammatory lid disorders should be studied in the future. Future confocal microscopy trials assessing the effect of different treatment protocols on MGs would also provide invaluable information.
